# Sexual Functioning among Married Iranian Women with Polycystic Ovary Syndrome

**Published:** 2014-11-01

**Authors:** Fatemeh Bazarganipour, Saeideh Ziaei, Ali Montazeri, Fatemeh Foroozanfard, Anoshirvan Kazemnejad, Soghrat Faghihzadeh

**Affiliations:** 1 Hormozgan Fertility and Infertility Research Center, Hormozgan University of Medical Sciences, Bandarabbas, Iran; 2 Faculty of Medical Science, Tarbiat Modares University, Tehran, Iran; 3 Mental Health Research Group, Health Metrics Research Center, Iranian Institute for Health Sciences Research, ACECR, Tehran, Iran; 4 Gametogenesis Research Center, Kashan University of Medical Sciences, Kashan, Iran; 5 Department of Biostatistics, Tarbiat Modares University, Tehran, Iran; 6 Faculty of Medical Sciences, Zanjan University of Medical Sciences, Zanjan, Iran

**Keywords:** Sexual Dysfunction, Polycystic Ovary Syndrome, Women

## Abstract

**Background:**

This study aimed to assess sexual functioning among women with polycystic ovary syndrome (PCOS) in Iran.

**Materials and Methods:**

A cross-sectional study was conducted to ascertain factors re- lated to sexual functioning in 300 PCOS patients attending to the private practice centers in Kashan, Isfahan Province, Iran, from May to October 2012. The Female Sexual Function Index (FSFI) was used to measure sexual functioning. Moreover, the socio-demo-graphic details and clinical information of PCOS including obesity, hirsutism, acne, mestrual cycle disturbances, infertility and endocrine profile were recorded for each patient.

**Results:**

Overall the prevalence of female sexual dysfunction (FSD) was 16.6%. In particular patients indicated poorer sexual functioning for the desire (48.3%) and the arousal (44.7%) subscales. Multiple logistic regression analysis suggested patients with lower educational level (OR: 2.94; 95% CI: 1.46-5.92) and irregular menstrual status (OR: 4.61; 95% CI: 1.93-11) were more likely to report sexual dysfunction.

**Conclusion:**

The findings suggest that desire and arousal were the most prevalent sexual disorders reported in this patient population. In addition, findings suggested that women with limited or no formal education and a history of menstrual irregularities were the most likely to report female sexual dysfunction. Further investigations are needed to examine female sexual functioning among women with PCOS, to educate their health care providers, and to develop therapeutic interventions.

## Introduction

Polycystic ovary syndrome (PCOS) is the most common endocrine disorder in women of reproductive age. It is estimated that 5 to 10% of women suffer from the disease (1). The symptoms typically associated with PCOS are irregular menstruation, hirsutism, obesity, infertility, anovulation and acne, leading to a significant reduction in female quality of life (QOL), marital maladjustment and impaired sexual functioning (2, 3).

Sexuality is an important and complex domain in QOL studies. Prevalence of female sexual dysfunction (FSD) may vary according to cultural, racial and health status. Impaired sexual functioning in women with PCOS has been often neglected or studied incidentally. Characteristics associated with PCOS may adversely affect sexual health. Women struggling with PCO, have reported feeling less attractive and having lower sexual satisfaction when compared to women without PCOS (4). Contrary to hypothesis stating that elevated androgen levels in PCOS increase female libido, women with PCOS have reported decreased sexual satisfaction and feeling less attractive (5-7). In one study findings suggested an elevated body mass index (BMI) did not affect sexual function or intercourse frequency, but a higher BMI resulted in a decrease in sexual satisfaction (8). Hahn et al. (2) reported that hirsutism decreased women sexual function more than obesity.

Studies examining the sexuality of patients with PCOS focused on the psychosexuality or subject’s sexual orientation (9-11). Since there are multiple factors that can impair the sexual function of these patients, it is essential to evaluate the importance of this problem and the main factors contributing to this disorder. In the Iranian population, there has been no study related to sexual functioning among women with PCOS yet. This study was designed to investigate whether clinical and hormonal characteristics in women with PCOS influenced their sexual functioning. The intent is to facilitate an understanding of the relationships between these variables and to guide interventions that might improve the sexual function of patients strugglingwith symptoms of PCOS.

## Materials and Methods

### Design and data collection

This was a cross-sectional study of women with PCOS who attended two private gynecology clinics in Kashan, Isfahan Province, Iran, from May to October 2012. The Ethics Committee of the Tarbiat Modares University approved the study. Patients with confirmed diagnosis of PCOS were invited to participate in the study. After explaining the study objectives, a written informed consent was obtained from all participants and they were then requested to complete the study questionnaires. Inclusion criteria were as follows: 15-40 years of age, married, Iranian, as well as having two of the following Rotterdam diagnostic criteria: i. polycystic ovaries being detected by ultrasound scan (presence of 12 follicles or more in one or both ovaries and/or increased ovarian volume >10 ml), ii. clinical signs of hyperandrogenism (hirsutism score based on hirsutism score greater than 7 or obvious acne) and/or an elevated plasma testosterone (testosterone >2. 0 nmol/l) (12-13), and iii. having an interval between menstrual periods >35 days and /or amenorrhea, defined as the absence of vaginal bleeding for at least 6 months (i.e.199 days) (14). Exclusion criteria were as follows: diagnoses of non-classical adrenal hyperplasia; thyroid dysfunction or hyperprolactinemia; communication concerns, specifically the inability to speak or listen attentively; previous or current psychiatric diagnosis or using psychiatric medications including antidepressants; and taking any prescription medication (except allergy medications and occasional pain medications) for at least three months before entering the study.

### Measures

#### Sexual function

Female sexual function was evaluated using a detailed 19-item questionnaire [the Female Sexual Function Index (FSFI)] described by Rosen et al. (15). This standardized questionnaire evaluates six domains of female sexual functioning during a four-week period that is identified as desire, arousal, lubrication, orgasm, satisfaction and pain during sexual intercourse. The domain of female sexual arousal disorder is assessed in terms of frequency, level, confidence and satisfaction with eight questions. It is further divided into two separate domains of lubrication (four items) and arousal (four items). These items assess both the peripheral (lubrication) as well as the central (subjective sexual arousal and desire) components. Other domains assessed include pain (three items), orgasm (three items) and satisfaction (three items). A scoring algorithm is applied to each domain and a composite score is obtained. Scores ranged for items 3-14 and 17-19 are 0-5, and for items 1, 2, 15 and 16 are 1-5. By adding the scores of the individual items comprising the domain and by multiplying the sum by the domain factor, individual domain scores are then obtained. Maximum scores for factors are as follows: 0.6 for desire, 0.3 for arousal and lubrication, and 0.4 for orgasm, satisfaction and pain. A total score is obtained by adding the six domain scores. The full-scale score range is from 2.0 to 36.0, with higher scores associated with a lower degree of sexual dysfunction. Women who scored <3.9 for all six domains are identified as sexual dysfunction. In this study, we used the cutoff points of the Persian version translated by Mohammadi et al. Therefore, a score <3.3 in the desire domain, score <3.4 in arousal and orgasm, score <3.8 in satisfaction and pain, score <3.7 in lubrication, and total score <28 were considered as female sexual dysfunction (FSD). Validity and reliability of Persian version of the questionnaire has been well documented (16). In order to carry out the test-retest reliability, a total of 30 patients, randomly selected from the original group, completed again the FSFI two weeks later, in the same manner as the first one. The test-retest reliability of the scale was estimated by intraclass correlation coefficient (ICC). The ICC was satisfactory (0.80, p<0.05).

#### Clinical symptoms of PCOS

1. Menstrual history: patients were asked to choose the best option indicating their menstruation interval during the preceding 12 months of the following category: <21 days, 21-34 days, 35-60 days, >199 days, and being variable.
2. Reproductive history: women were asked to categorize their reproductive history based on the following criteria: i. having been pregnant: all births, no losses; ii. having been pregnant: some births, some losses; iii. having been pregnant: no births, all losses; and iv. never being pregnant.
3. BMI: weight and height were calculated by the following formula for all participants, weight/ height squared (kg/m2).
4. Body hair: clinical assessment of hirsutism was determined using the Ferriman-Gallwey scoring system (F-G score). Nine body sites (the upper lip, chin, chest, upper back, lower back, upper abdomen, lower abdomen, arm, and thigh) were graded from 0 (no terminal hair) to 4 (severe hirsutism). Scores can range from 0 to 36. A score of 7 or above was considered positive for hirsutism (17).
5. Acne: acne was determined using the Global Acne Grading System (GAGS). The GAGS considers six locations on the face and chest/upper back. The borders on the face are defined by the hairline, jawline, and ears. The score of each location is a factor presenting affected surface area as well as distribution and density of pilosebaceous units. The chest and upper back are also included because their involvement is critical in order to assess the severity of acne and to decide on treatment option. The score of each location is separately determined in a 0-4 point scale that means the sum of scores belonging to a location (18).

#### Socio-demographic status

The study used years of formal education as a measure of socioeconomic status that was categorized into five levels: no education, first level (1 to 5 years), second level (6-9 years), third level (10-12 years) and fourth level (more than 12 years). Different studies from Iran showed that education could be a good proxy measure for socioeconomic status of Iranians (19).

### Laboratory measures

An overnight 8-hour fasting venous blood sample was obtained from each subject on the second or third day of their spontaneous or progesterone-induced menstrual cycles. Serum total testosterone (TT), sex hormone-binding globulin (SHBG), follicle-stimulating hormone (FSH), and luteinizing hormone (LH) were concomitantly assessed in all participants by ELISA (DRG Instruments GmbH, Marburg, Germany). TT and SHBG were used to calculate the free androgen index (FAI) as TT (nmol/l)/ SHBG (nmol/l) ×100, suggesting to be a useful indicator of abnormal androgen status (20).

### Data analysis

Data are presented as number (%), unless otherwise indicated. To explore the association between the socio-demographic and PCOS characteristics with sexual function (Table 1), the method of multivariate logistic regression analysis was applied. Odds ratios (ORs) and 95% confidence intervals (CI) were calculated. Independent variables included the PCOS characteristics that were dichotomized, converted and coded as dummy variables. For example, menstrual cycle was converted to 1 that indicates amenorrhea, oligomenorrhea, polymenorrhea, or being variable, or to 0 that indicates the remaining, while the variables remaining in the model were reported. A value of p<0.05 was accepted as significant. Statistical analysis was performed using Statistical Package for the Social Sciences 15.0 (SPSS Inc., Chicago, IL, USA).

**Table 1:** Demographic and (bio) clinical characteristics of PCOS patients


Demographic data	

Age (Y)*	26.56 ± 4.44
Education**	
The first level	32(10.7)
The second level	50(16.7)
The third level	126 (42)
The fourth level	92(30.7)
Duration of marriage*	10.02 ± 4.20
Parity*	0.51 ± 0.77
**Clinical**	
Hirsutism score *	6.7 ± 5.73
Acne score *	10.54 ± 7.26
Interval between menstruation (days)**	
<21	8 (2.7)
21-34	109 (36.3)
35-60	19(6.3)
>199	31(10.3)
Variable	133 (44.3)
Reproductive history **	
Never being pregnant	193 (64.3)
Having been pregnant: all births, no losses	32(10.7)
Having been pregnant: some births, some losses	17(5.7)
Having been pregnant: no births, all losses	58(19.3)
BMI (kg/m^2^)**	
<25	130 (43.3)
25-30	120 (40)
>30	50(16.7)
**Endocrine**	
LH (IU/l)*	8.28 ± 6.16
FSH (IU/l)*	6.09 ± 4.42
Testosterone (nmol/L)*	1.24 ± 0.23
SHBG (nmol/L)*	55.57 ± 43.87
FAI*	10.21 ± 34.45


PCOS; Polycystic ovary syndrome, BMI; Body mass index, LH; Luteinizing hormone, FSH; Follicle-stimulating hormone, SHBG; Sex hormone-binding globulin, FAI; Free androgen index, *; Mean ± SD and **; N (%).

## Results

### Socio-demographic characterize and clinical symptoms

In all, 300 women with PCOS were included in the study during the six-month enrollment. The mean (SD) age of patients was 26.5 (4.44) years. The majority of women had education beyond high school (72.7%, n=218). More than two-thirds of patients had never been pregnant and had not successfully carried a pregnancy to term (n=251), of whom most reported having abnormal menstruation (n=191). Our inclusion criteria were FG score more than >7 and testosterone level >2. However, our findings showed that the mean values of FG score and testosterone were 6.7 and 1.24, respectively. According to Rotterdam criteria, having two of the diagnostic criteria is enough. In other word, if a patient complains of irregular menstrual cycles and her sonography results also indicates polycystic ovary, it is considered as a common case of PCOS. For this reason, the mean scores of hyperandrogenism are lower than inclusion criteria. Socio-economic and clinical characteristic of the patients are presented in table 1.

### FSFI subscale’s scoress

**Fig 1:**
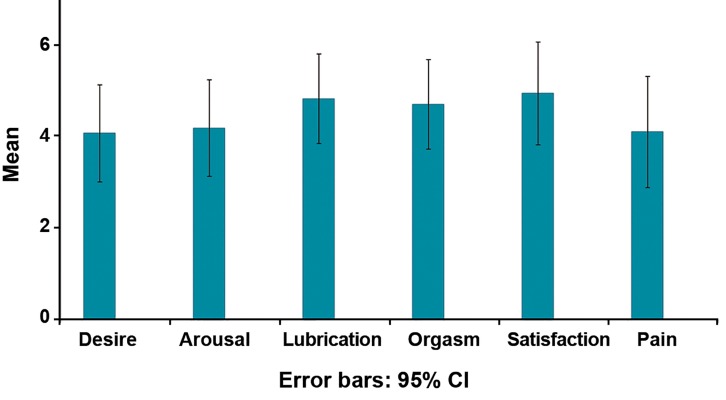
Mean FSFI scores of subscales in PCOS patients.

Fig 1 presents a summary of the mean scores of the six subscales, indicating FSFI, while the overall prevalence of FSD is 16.66% (n=50/300). The items are arranged from highest to lowest scores as follows: i. desire (48.3%, n=145/300), ii. arousal (44.7%, n=134/300), iii. pain (39%, n=183/300), iv. lubrication (21.3%, n=64/300), v. orgasm (15%, n=45/300), and finally vi. satisfaction (13%, n=39/300).

### Factors contributing to sexual dysfunction according to logistic regression

Multiple logistic regression analysis suggested a positive association between FSD and menstrual disturbance (OR: 4.61; 95% CI: 1.93-11). In other word, women with menstrual irregularities reported higher levels of sexual dysfunction when compared to PCOS women with regular menstruation cycles. Moreover, FSD was significantly higher in the presence of low level of education (OR: 2.94; 95% CI: 1.46-5.92, Table 2).

**Table 2:** Logistic regression analysis including socio-demographic and clinical symptoms predicting FSFI score among PCOS patients


Independent variables†	OR (95% CI)	SE	P value

**Education**	2.94(1.46-5.92)	0.35	0.002
**Menstrual**	4.61(1.93-11)	0.44	0.001


†; Age, F/G and acne scores, duration of marriage, parity, endocrine profile and BMI were included in the regression analysis as continuous variables and other variables were used as dummy variables. Only significant results are presented. FSFI; Female sexual function index, PCOS; Polycystic ovary syndrome, CI; Confidence interval and OR; Odds ratio.

## Discussion

Since PCOS often manifests itself through marriage and having sexual activity, its psychosexual implications are found to cause profound emotional distress in affected women (21). The concept of sexual problems has not been discussed in Iranian PCOS patients. The objective of this study was to further examine the impact of PCOS on female sexual function in an Iranian population sample and to identify potential demographic and patient-related risk factors for FSD.

Given the effect of PCOS on women’s physical health and emotional well-being, it is undoubted that a substantial proportion of patients reported sexual impairment and problems. In our study, the overall prevalence of FSD was 16.66%. The most significantly influenced domains in these participants were in line with the results of Aslan et al. (22) about desire and arousal. Sexual arousal as a separate component of the sexual response cycle was first recognized by Kaplan (23), while arousal problems are often considered to be attributed to inhibited desire that may occur independently (24). The changes that occur in a woman’s physical appearance as a result of PCOS, particularly hirsutism, acne and obesity, along with menstrual irregularity and infertility, have been found to be a leading cause of psychological morbidity (9, 25-31). Moreover, psychological inhibition may result in inadequate vaginal lubrication and cause coital pain. Almost 39% of the women reported having pain during intercourse, while sexual aversion, inconvenient relationship effects, and development of additional sexual dysfunction were also reported. The pain may prevent intromission, while considering to prevalence of infertility in this population, the importance of this issue is obvious.

In this study, 15% of the studied sample reported orgasm disorder. Depression, poor body image and low self-esteem were frequently seen in PCOS patients (32-36), indicating as a main cause of orgasm disorders in these women. In our study, 13% of women experienced a lack of satisfaction with their sexual relationship. In a study by Hahn et al. they assessed the quality of life (QOL), psychological well-being and sexual satisfaction of 120 patients with the diagnosed polycystic ovary syndrome and showed a subjective deterioration of general well-being as well as an increase of psychological disturbances and sexual problems in women with PCOS (2). In another study by Elsenbruch et al. (9) they observed that the manifestations of PCOS, such as infertility, hirsutism, acne, and obesity, lead to reduction in QOL and to serious limitation of sexual satisfaction.

In the present study, we further determined factors that may influence sexual function in PCOS women. Of the socio-demographic data analyzed, education level showed strong correlation with the likelihood of FSD. Lower educational levels are positively associated with the presence of sexual dysfunctions, as also shown by similar findings of studies conducted in Turkish, Africa, and USA (37-41).

In the current study, women with menstrual irregularities reported higher levels of sexual dysfunction when compared to PCOS women with regular menstruation cycles. A negative effect of menstrual problems on the quality of life of patients has been previously discussed by other authors as well (28). Menstrual irregularities can have important social consequences, especially for Muslim women. For example, it is forbidden for a menstruating woman to perform many religious activates, like prayer; therefore, prolonged bleeding disrupts household patterns in such a way that family and community members may become aware of a woman’s situation if her period persists for more than the expected number of days (42, 43). Menstrual irregularities may also have adverse consequences for women’s intimate relations and for other aspects of their reproductive and general health. For example, in Islam, man is forbidden to have intercourse with his wife during her menses as in Judaism and Zoroastrianism.

Surprisingly, we did not find any association between FAI levels and FSFI domain scores. Our results are consistent with the findings by Davis et al. (44), indicating no association between low sexual domain scores and low free testosterone serum levels. Thus the hyperandrogenism characteristic of PCOS does not predict satisfactory sexual functioning in our sample, even though endogenous testosterone is known to play an important role in this regard in normal women (45, 46). In contrast, a previous study in non PCOS women showed that a positive correlation between higher testosterone level and ability to achieve an orgasm in women of reproductive age (47). It is possible that a positive association between androgen levels and satisfying sexual functioning is masked by the effects of the PCOS phenotype on self-esteem, which is crucial to sexual functioning. It is well known that the hyperandrogenic phenotype deleteriously affects the emotional condition of patients with PCOS. Such symptoms may be associated with reduced sexual and body image satisfaction (30, 48). In addition, a negative self-image, a higher BMI, depression, sexual dysfunction, reduced lubrication, and lower sexual excitement have been reported in women with higher testosterone levels (2).

As FSD is known as a common health problem in PCOS women, some controversy exists concerning the prevalence of FSD, while unique national, religious and cultural variations may contribute to risk factors of FSD. However, a thorough evaluation between different studies is affected by the lack of a uniform validated FSD questionnaire, setting, definition of FSD characteristics of the study population and the method of evaluation. A main methodological problem is use of the internationally accepted FSD questionnaires. At present, the FSFI is the most commonly used FSD questionnaire that has acceptable reliability and validity (16). Strengths of the current study were excellent response rate and verification of findings by gynecology physicians. We evaluated sexual function in married Iranian women with PCOS using a standardized questionnaire and a collection of questions designed for studying characteristics of PCOS, specifically. Despite the importance of the present findings, this study has some limitations. Firstly, we were not able to include a matched control group because of difficulties in screening and diagnosis of PCOS, as discussed above.

The current results, therefore, are applicable to identify the differences within the PCOS population. Secondly, the data were collected from a married Iranian patient sample; therefore, the findings should not be extrapolated to the general population and need to be studied in larger sample size. Thirdly, we were not able to determine the direction of causality between our variables. Moreover, all included patients in this study were married for cultural reasons (sex and infertility) in Iran. Additional prospective researches are needed to investigate the link between infertility and FSD and to determine the relationship between other known risk factors and sexual function.

## Conclusion

This is a pioneer study in Iran investigating sexual problems in women with PCOS reporting sexual dysfunction, accounted as one-fifth of total participants. Desire and arousal disorders were the most common sexual dysfunction reported by Iranian women with PCOS. Our finding revealed that subjects with limited or no formal education and a history of menstrual irregularities reported greater sexual dysfunction using the FSD scale. In order to determine the causes of FSD, the topic needs further exploration involving intervention at regular health care visits. Clinician should consider religious and cultural background of their patients, especially in view of the factors influencing FSD.
